# Sociodemographic Determinants and Students’ Perception Regarding Legislative Changes

**DOI:** 10.25122/jml-2019-0082

**Published:** 2020

**Authors:** Simona Parvu

**Affiliations:** Hygiene and Medical Ecology Department, “Carol Davila” University of Medicine and Pharmacy, Bucharest, Romania

**Keywords:** health legislation, legislation changes, students’ perception, socio-demographics, gender difference

## Abstract

The frequency of legislation changes and their utility is a subject of interest and always under debate with pro and contra arguments. In the current study, the perception of students regarding medical legislation changes was analyzed. In general, no statistically significant differences were found between the sub-groups of students, thus underlining a unitary perception. The general perception is that changes in legislation are made too often. Moreover, only a small part of the analyzed group agrees on the utility of these changes. Finally, it seems that income is the main driver for students as future employees in the medical system. Also, significant differences between males and females’ perceptions are emphasized, and legislation changes should take into account the primary drivers. Other issues, such as corruption, stability, and performances should be the key points in legislation changes. These results should be a challenge for all stakeholders, in particular for policymakers.

## Introduction

It is known that the health system in Romania has undergone significant changes since 1989, which aimed to move from a centralized and exclusively funded system from the state budget revenues to a decentralized system, financed mainly by insurance contributions [1].

In 2008, a committee organized by the Presidential Administration noted that there is “poor management of health information with respect to the existence of several parallel systems, coordinated and controlled by different owners” [2]. This finding comes as the reforms envisaged the National Health Insurance Agency, the change of the form of organization of hospitals, and the emergence of the contractual relations between providers and insurance funds (public or private). Now, we can say that after the decentralization has resulted in more financial flows, but without a clarification regarding the responsibility to cover the need for investments in the health sector and service providers and by diversifying the state's ability, the expenditure was exceeded [3]. Therefore, in 2005, the National Health Insurance Agency introduced ceilings and penalties for the suppliers who failed to meet the budget.

One specific issue is the reform of the system image created by the media channels and politicians involved in the field. A survey of the Romanian Institute for Evaluation and Strategy (IRES) from 2010 reveals dissatisfaction with the quality and variety of food, comfort, and hygiene in the hospital [4]. However, the majority of respondents (77%) were satisfied and very satisfied with the care provided. Another theme of dis/information is the financial burden of people employed to cover health services further. Redefining the basic package and the introduction of co-payment could not be evaluated adequately as a financial impact, considering the lack of information on costs of voluntary policies and services covered.

In chronological order, the health reform processes that took place between 1990 and 2012 included the following aspects:

• remuneration of the medical personnel, a project developed in 2011;
• reviewing the length of hospitalization for various diagnostic groups;
• increasing tax-deductibility and voluntary private health insurance;
• change of management in the conditions of decentralization of 373 hospitals under the subordination of local authorities, only 62 hospitals remaining subordinated to the responsible ministry;
• closure of 67 hospitals, the majority in rural areas;
• implementation of a unique integrated system to transmit information flows between healthcare providers and insurance funds in real-time;
• implementation of the electronic prescription and the health insurance card.

Significant changes in the health system were made without being able to estimate the impact on the system. This impact was subjected to analysis in medical sciences, representing a national security interest as well. For example, Popa underlines that we have articulated scientific literature related to health system reform and decentralization [5]. The author appreciates the Romanian health care reform in the context of the reform of all health systems in the formerly communist area, where decentralization is regarded rather as a means of achieving health reform and not an end in itself. “Decentralization is part of the trend of wider empowerment of local communities for various public issues. Decisions and programs related to education, funding, or health are transferred to more countries and ever more frequently, locally. Local authorities and community members are placed more frequently to work together, collaborate, participate and, finally, to come to understand that the health of that community is a matter of common interest and it dependents on the actions, programs, decisions at the local level”, says the author.

However, there is still no comprehensive analysis of specific segments or of the national health system to show which were the visible or hidden effects of successive reforms, and which were the advantages or disadvantages. From this perspective, it is evident that estimates of future strategies are based on quantified results, which would increase the risk of successful implementation of future actions and acceptance of quality indicators.

Through a previous study conducted a few years ago, we investigated the perception of students enrolled in Romanian universities with medical programs, the impact of legislative changes on the quality of health care, but also how they perceive personal career development in the context of the mentioned changes [6]. The study had the following objectives: establishing a representative sample of the studied population - I - VI year medical students; development and validation of a questionnaire to investigate both students’ knowledge on health legislation and how they obtain the information on this topic; interpretation of results, conclusions, and recommendations.

The hypothesis that started the study was based on the perception that public health policies do not meet the health needs and are not supported by evidence; also, the results of implementing legislative and organizational changes are not assessed to measure the impact on the quality and performance of health services (assumptions: health reforms and health policies are not based on evidence and poor management of health information mainly inadequate data collection causes inability to document decisions on priority areas of intervention). Repeated legislative changes were based on somewhat different visions of reforms, without a holistic, coherent vision.

A similar vision regarding the legislative changes was also found by Vogenberg [7]. Moreover, he also emphasizes instability and uncertainty as being the main effects of frequent changes.

It is known already that there is a real crisis in Romania regarding the number of doctors of various specialties, which have to provide medical assistance at the optimal level [8]. This crisis is shared by other countries, but the causes may be different, depending on demographic and socio-economic characteristics, in particular, specific morbidity [9]. Details about this deficit are numerous, and governmental structures should be concerned to identify measures to urgently correct factors that may adversely affect the efficiency of health systems in each country at a European level [10].

The debates regarding the legislation changes [10], and different stakeholder perceptions are still a challenge for both practicians and academics. Then, feedback from students from the medical field, as current stakeholders or potential stakeholders in the future, regarding the subject of legislative changes, may complete the overall image of perceptions.

For higher accuracy of any analysis, data/perceptions of the analyzed phenomenon/activity should be collected from all stakeholders directly and indirectly. Here, analyzing the impact that legislative changes (“reforms”) have produced on the national health system rests even on the perception of future skilled resources in the field, namely the medical students, both on the current reality of health care facilities and the future needs of the curriculum.

## Material and Methods

The target population is represented by medical students. The sample was calculated based on the information posted on official websites and the number of students enrolled in medical programs in five universities.

The development of the review questionnaire aimed at investigating students’ perceptions of the impact of legislative changes on the quality of healthcare services and their information sources on this topic. A questionnaire with 23 items, which reported data on gender, age, year of study, sources of information on health legislation, the view on the impact of legislative changes on career and system health and medical woes opinion and perspective on the profession was built and validated to investigate the knowledge and students’ opinions.

The questionnaire was distributed online with the help of teachers from the faculties of medicine and completed voluntarily through Google-Form. Since the answer is voluntary, with random selection, the sample is one of convenience.

According to the information displayed on the official website of the Faculty of Medicine from Bucharest, Craiova, Targu-Mures, Sibiu and Arad, about 14,029 students are attending the six-year program. The sample volume for a representative sample was estimated using Open-Epi, web-base open-source interface [11] under the following hypothesis:

• target population volume (N=14,029);
• hypothesized proportion for outcome factor in the population p=0.5, while this implies the maximum variance σ(p)=p(1-p), assuming in this way the highest incertitude;
• margin of error Δ(p)= +/- 5p.p.;
• assumed type I error = 0.05 => Confidence level P=0.95;

In these conditions, the estimated sample volume is 374 units (students).

Besides basic descriptive statistics, differences between the groups were tested using Pearson's correlation coefficient χ2= n∑[(Oi-Ei)2/Ei], relation derived from McHugh [12]. The Oi and Ei denote the observed and expected shares (%) for certain sub-groups defined by the counter i. The null hypothesis was rejected if the p-value (type I error) was less than 0.05.

Regarding the utility of legislation changes, in order to increase the discrepancies between groups and to keep a minimum sub-sample volume (e.g., n>5), the utility was measured using a 5-point Likert scale with 3 = neutral point. In the analysis, the neutral category (3) was ignored, and the respondents rated their level of agreement regarding the usefulness of the changes by giving 4 or 5 points) or disagreement by giving 1 or 2 points.

In the cases of legislation changes which may motivate or demotivate the employees or potential employees, a dummy variable was defined as follows:

• students for which salary/income is the first/most important/primary motivator.
• students for which other factors, such as hospital conditions, work-place stability, and others, are the main motivators.

Using the Chi-squared Statistic and/or Yule's Q in two by two tables [13], the association between students’ characteristics and dummy motivating factors was analyzed. The results are given in the next paragraph.

## Results

The random sample has 87.0% of the subjects aged under 25, and 65.2% of the total are female. Most of the students (60%) were in their fifth or sixth university year. Most students reside in the university center where they study: 54,3%.

**Figure 1:**
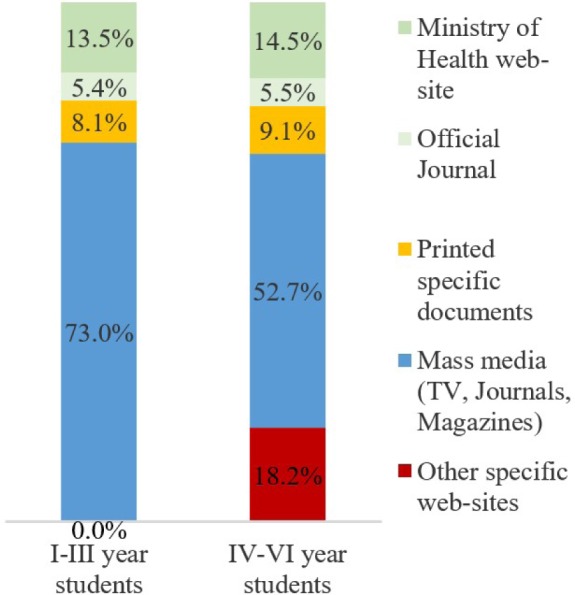
Sources of information.

Students participating in the study regarding health policy measures inform themselves differently regarding frequency, the medium, the subject of interest, or the time devoted to this activity (Figure 1). Students in later years of study tend to be less individually informed. 56.4% of the students in the IV-VI years of study inform themselves annually or less, while more than half of students in the I-III years of study inform themselves with a higher frequency, at least once per semester. This reversal of interest can be derived precisely from the frequent legislative changes, students in later years of study may consider that they will comply with the legislative requirements at the moment they will start their professional career, which would justify a postponement of the information required by that point.

Based on the feedback, the most important source of information is the media (TV, newspapers/magazines). This is accessed by over half of the students. The second source of information, as frequency, for students in the IV-VI years of study, is given by more specialized sites, such as avocat.net or lex.ro. It is worth noting that the website of the Ministry of Health is the last source of information as frequency.

Unexpected, although the individual information is scarce in their case, a higher share of participation in courses on the subject of legislation in the field meets, however, students in later years of study (20%) higher in female students (15.0%) than male students (9.3%). According to the Chi-squared test results, there is no statistically significant difference between young students (I-III year) and experienced students (p>0.05).

It is also of interest that students in Targu-Mures and Craiova, questioned within the study, did not participate in specific courses about legislation, and the share of students in Bucharest in such activities is minimal.

**Figure 2:**
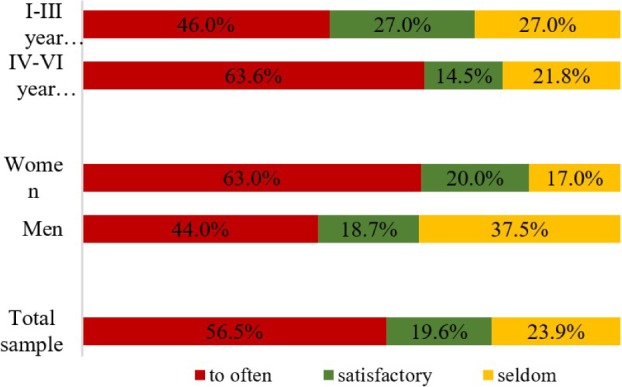
Opinion on the frequency of legislative changes.

The general opinion is that the legislation is too frequently amended (Figure 2). The percentage of female students who agree is 63%. The same opinion is shared by only 44% of male students. The Chi-squared test did not identify a significant difference between the groups at the 0.05 level. However, in the case of gender comparison, the type I error is very close to the widely accepted level (p=0.07).

**Figure 3:**
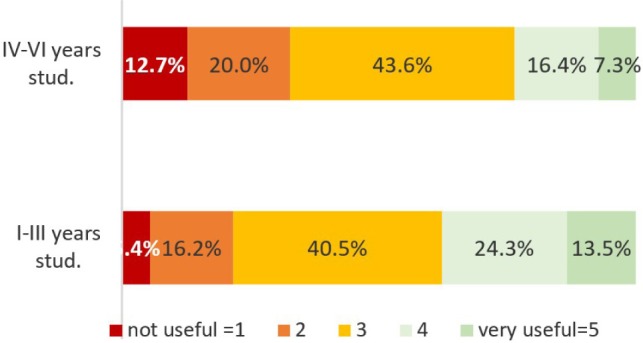
The utility of legislative changes.

Students have a rather neutral or negative opinion concerning the utility of changes in legislation (Figure 3). Less than a quarter of the students during the IV-VI years of study considered the changes useful, and about 37% students of years I-III consider them useful or very useful. When the statistical analysis was made between the subgroups of students, comparing young vs. experienced students and students who agree or disagree with legislation changes, the results showed no significant difference (p>0.05).

It is significant that, although formally, a legal act/decision submitted for decisional rules of transparency should be easily accessible, including the media, the majority of respondents do not know the procedure for consultation of a draft legal act. All who know the transparent decision procedure considers it very good. However, since the media was the main information source, it is confirmed that the main actor in promoting legislative changes (the ministry), which must ensure compliance with the transparency rules in decisions, failed to be considered a center of information of interest to the respondents.

**Figure 4:**
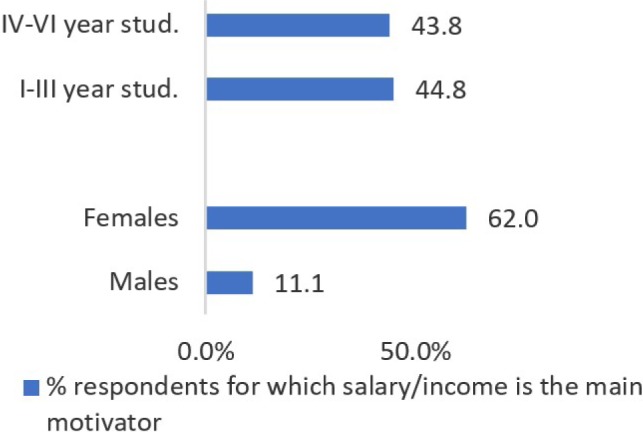
Respondents considering the salary/income as the primary motivating factor.

On average, almost 50% of students would like to see in the legislation changes some paragraphs which ensure a minimum decent wage/income, as for them, this is the most important motivator (Figure 4). When Yule's coefficient was used to test the statistical difference between groups, it was found that no significant differences are perceived between the year group of the enrolled students, but a statistically significant difference (p<0.01) was found between males and females. It seems that income/wage is more important for females (62% of them), while for most of the males (89%), other factors such as hospital equipment availability or job stability are the primary drivers of motivation.

Besides the measurable results of the survey, the respondents indicated which would be the main problems related to the poor performance of the health system. Overall, these concerns and the need for intervention are exposed below:

• stop delaying the creation of a practical framework for implementing the legislation;
• eliminating the bureaucracy and ambiguity of laws, corruption and indiscipline;
• adequate allocation of resources in hospitals and increasing the equipment level, especially in the rural areas;
• allocating more funds to the health system;
• eliminating the sudden changes/fluctuations of rules concerning the different parties in power;
• the lack of correct information;
• the lack of investment;
• the lack of jobs;
• the lack of a platform for a paid internship for students (engineers go into factories, but young doctors do not have where to go);
• the lack of standardized protocols for each facility;
• eliminating the situations where the National Health Insurance Agency does not fund all investigations/interventions required to diagnose or prescribe reimbursed treatment;
• establishing a medium-term strategy and long-appropriate vision;
• implementing changes that will increase environmental health standards.

## Conclusions

The information sources of students participating in the study regarding health policy measures differ. However, students in their final years tend to be less individually informed as a result of the frequent legislative changes because they consider that they will comply with the legislative requirements the moment they start working as doctors, which would justify a postponement of the information required by that point.

Students use the media as an information source (TV or press) and specialized legislative sites, while the Ministry of Health website is the last source of information for all students. Should the representative authority in this field promote different communication models or organize and distribute more “friendly” the information of interest, including for future doctors?

The study showed an average level of student interest for changes in legislation, but the general opinion is that the legislation is too frequently amended.

There are universities where no student attended a legislation class, and in other centers, the participation is represented by a modest percentage.

In terms of the utility of the legislative changes, students have a neutral or negative opinion. It would seem useful to include in the student's medical curricula, especially in their final years, courses on health legislation, regulations in cases of malpractice, and the rights and responsibilities of a doctor in Romania.

No statistical differences were found between the subgroups regarding age, experience, or gender. The absence of differences shows a unitary opinion regarding the legislation changes.

The income/wage represents the main motivating factor for students. A statistically significant difference was found between males and females in this sense. Females are somewhat more interested in a minimum income/wage, while males are more interested in job stability, device availability, and others. Thus, legislative changes should provide adequate measures considering these opinions, and stakeholders should take the challenge.

## Conflict of Interest

The authors declare that there is no conflict of interest.
